# Paradoxical psoriasis induced by TNF‐α blockade shows immunological features typical of the early phase of psoriasis development

**DOI:** 10.1002/cjp2.147

**Published:** 2019-10-29

**Authors:** Luca Fania, Martina Morelli, Claudia Scarponi, Laura Mercurio, Fernanda Scopelliti, Caterina Cattani, Giovanni Luca Scaglione, Tiziano Tonanzi, Maria Antonietta Pilla, Gianluca Pagnanelli, Cinzia Mazzanti, Giampiero Girolomoni, Andrea Cavani, Stefania Madonna, Cristina Albanesi

**Affiliations:** ^1^ Laboratory of Experimental Immunology and 1st Dermatology Division IDI‐IRCCS Rome Italy; ^2^ Section of Dermatology, Department of Medicine University of Verona Verona Italy; ^3^ Istituto Nazionale per la promozione della salute delle popolazioni Migranti ed il contrasto delle malattie della Povertà INMP Rome Italy; ^4^ Laboratory of Molecular Oncology, “Giovanni Paolo II” Foundation Catholic University of Sacred Heart Campobasso Italy

**Keywords:** psoriasis, hidradenitis suppurativa, anti‐TNF‐α therapy, paradoxical psoriasis, skin inflammation, innate immunity, type I IFN, lymphotoxin

## Abstract

Immunomodulation with anti‐TNF‐α is highly effective in the treatment of various immune‐mediated inflammatory diseases, including hidradenitis suppurativa (HS). However, this may be responsible for unexpected paradoxical psoriasiform reactions. The pathogenic mechanisms underlying the induction of these events are not clear, even though the involvement of innate immune responses driven by plasmacytoid dendritic cells (pDC) has been described. In addition, the genetic predisposition to psoriasis of patients could be determinant. In this study, we investigated the immunological and genetic profiles of three HS patients without psoriasis who developed paradoxical psoriasiform reactions following anti‐TNF‐α therapy with adalimumab. We found that paradoxical psoriasiform skin reactions show immunological features common to the early phases of psoriasis development, characterized by cellular players of innate immunity, such as pDC, neutrophils, mast cells, macrophages, and monocytes. In addition, IFN‐β and IFN‐α2a, two type I IFNs typical of early psoriasis, were highly expressed in paradoxical skin reactions. Concomitantly, other innate immunity molecules, such as the catheledicin LL37 and lymphotoxin (LT)‐α and LT‐β were overproduced. Interestingly, these innate immunity molecules were abundantly expressed by keratinocytes, in addition to the inflammatory infiltrate. In contrast to classical psoriasis, psoriasiform lesions of HS patients showed a reduced number of IFN‐γ and TNF‐α‐releasing T lymphocytes. On the contrary, IL‐22 immunoreactivity was significantly augmented together with the IL‐36γ staining in leukocytes infiltrating the dermis. Finally, we found that all HS patients with paradoxical reactions carried allelic variants in genes predisposing to psoriasis. Among them, SNPs in *ERAP1*, *NFKBIZ*, and *TNFAIP* genes and in the *HLA‐C* genomic region were found.

## Introduction

TNF‐α blockers are efficaciously utilized in the treatment of various immune‐mediated diseases, such as psoriasis, rheumatoid arthritis and, more recently, hidradenitis suppurativa (HS) 1, 2. However, cutaneous reactions, such as eczematous and psoriasiform lesions, and other side effects have been reported 3, 4, 5. Some of these adverse reactions are considered as paradoxical effects and, in particular, 2–5% of patients treated with TNF‐α antagonists develop paradoxical psoriasiform skin lesions 6, 7, 8, 9. These reactions may require the interruption of the imputable drug, and no other biologics are approved for diseases like in HS. Therefore, it is important to understand the pathogenesis of these reactions, and a possible genetic susceptibility should be examined in these patients.

Psoriasis is a chronic inflammatory skin disease mediated by autoreactive T cells, which produces epidermal keratinocyte hyperproliferation with aberrant differentiation and senescence 10, 11. Early upstream events occurring in psoriasis include induction of innate immunity responses, primarily depending on keratinocytes activated by mechanical trauma, pathogens or drugs. At this initial phase, keratinocytes establish innate immunity circuits involving neutrophils, mast cells and macrophages and, importantly, enable plasmacytoid dendritic cell (pDC)‐ and myeloid DC (mDC)‐driven responses 12, 13, 14. Local production of type I IFN, as well as TNF‐α and IL‐6, by pDC and mDC unleashes adaptive immune responses, with expansion of T lymphocytes, typically Th17 and Th22 in the initial phase and Th1 cells during the chronic phase of the disease 10, 11, 15. Hence, lymphokines released in skin lesions, in particular IL‐17, IL‐22, and IFN‐γ, further amplify local immune responses 10, 16, 17, 18. Chronic immune responses are absent in paradoxical psoriasis induced by TNF‐α blockers, with innate inflammatory processes predominant and not followed by expansion of autoreactive T cells 19. These processes are concomitant with dermal accumulation of immature pDC and type I IFN overexpression 19.

Several studies have shown that intrinsic defects in genes controlling T‐cell commitment and keratinocyte inflammatory activation are associated with psoriasis 20, 21. Among them, the *HLA‐Cw6* allele represents the strongest genetic risk variant associated with psoriasis 22. The *HLA‐Cw6* haplotype might influence antigen presentation and immune responses, especially when associated with variants in the *ERAP1* gene, encoding an aminopeptidase involved in the formation of the peptides loaded on MHC class I molecules 23. Interestingly, a number of allelic variants were found in genes encoding signal transducers associated with IL‐17 or TNF‐α, such as *NFKBIZ* and *TNFAIP3*, encoding IKBζ and A20 proteins, respectively 24. Both IKBζ and A20 proteins regulate IL‐17‐ and TNF‐α‐induced molecular signaling, being an activator and a negative regulator of NF‐κΒ respectively 25.

Here, we report the immunological and genetic profiles of HS patients who developed psoriasiform reactions following anti‐TNF‐α therapy with adalimumab. We found that paradoxical psoriasiform skin predominantly shows immunological features common to early psoriasis, characterized by a massive infiltrate of innate immunity cells and local overproduction of innate immunity molecules. In contrast to classical psoriasis, psoriasiform lesions showed an increased number of infiltrating IL‐22^+^ leukocytes. Finally, we found that all the HS patients with paradoxical reactions carried allelic variants in genes predisposing to classical psoriasis, including SNPs in the *HLA‐C* genomic region.

## Material and methods

### Patients and samples

Three patients affected by HS, who developed psoriasiform skin lesions after treatment with adalimumab **(**40 mg, weekly), and three patients affected by classical plaque‐type psoriasis (PASI 8, 11.5, and 10) were included in the study. Clinical data, as well as skin biopsies and blood, were collected from patients with the permission of the IDI‐IRCCS Local Ethics Committee (Prot. CE 475/2016).

8‐mm skin biopsies were taken from psoriasiform lesions arising in HS patients or from 1.5‐month old psoriatic plaques. Biopsies were divided into two parts for immunohistochemistry and isolation of skin‐infiltrating T lymphocytes. A 2‐ml sample of peripheral blood was used to extract DNA, whereas a 20‐ml sample was used to isolate peripheral blood mononuclear cells (PBMCs).

### Immunohistochemistry

5‐μm paraffin‐embedded skin sections were stained with H&E or processed for immunohistochemistry. The primary antibodies used were as follows: anti‐BDCA2 (DDX0043‐TDS, Dendritics, Lyon, France), anti‐CD15 (#347420, BD Biosciences, Milan, Italy), anti‐IL‐17A (#AF‐317‐NA, R&D Systems, Abingdon, UK), anti‐lymphotoxin (LT)‐α (#SC8302, Santa Cruz Biotechnology, Dallas, TX, USA), anti‐IL‐22 (#NB100‐733, Novus Biologicals, Centennial, CO, USA), anti‐IFN‐κ (#H00056832‐M01, Abnova, Taiwan), anti‐CD117 and anti‐CD11C (#MONX10234 and #MON3371, Monosan, Uden, Netherlands), anti‐CD68 and anti‐CD3 (#P02246IT and #A0452, Dako, Glostruk, Denmark). The following antibodies came from Abcam (Cambridge, UK): anti‐IFN‐γ (#AB218426), anti‐IL‐36γ (#AB156783), anti‐IFN‐β1 (#AB180616), and anti‐LT‐β (Cat#AB64835). Immunoreactivities were developed using the 3,3′‐diaminobenzidine *HRP* substrate. Sections were counterstained with Mayer's hematoxylin.

### T‐cell isolation from skin biopsies and FACS analysis

T lymphocytes were isolated from skin biopsies as previously described 26. After 4–7 days, cells that had emigrated from biopsies were collected and characterized phenotypically. Lymphocytes were stained with the following monoclonal antibodies (mAbs): anti‐IFN‐γ‐FITC (#B27), ‐CD4‐PE (#RPA‐T4), ‐CD8‐PeRcP (#SK1), ‐CD3‐FITC (#HIT3a) (BD Biosciences); anti‐TNF‐α‐FITC (#6n1E7, Miltenyi Biotec, Bergisch, Germany), ‐IL‐17‐PE (#eBio64DEC17, EBiosciences, Frankfurt, Germany); anti‐IL‐22‐PeRcP (#142928, R&D Systems). Acquisitions were performed using an Attune Nxt (Life Technologies, Carlsbad, CA, USA). Analyses were performed using Flow logic software (Miltenyi Biotec).

### Real‐time PCR analysis

Total RNA was extracted from skin biopsies using RecoverAll Total Nucleic Acid Isolation (Life Technologies), and analyzed by real‐time PCR 27. The primer sets were as follows:

IFN‐α2A, 5′TCTGCTATGACCATGACACGAT3′/5′CAGCATGGTCCTCTGTAAGGG3′;
IFN‐β, 5′CAGCAATTTTCAGTGTCAGAAGC3′/5′TCATCCTGTCCTTGAGGCAGT3′;
IFN‐λ1, 5′AGGCTTCTCCAGGTGAGGGA3′/5′TCCAGGACCTTCAGCGTCAG3′;
IFN‐λ2, 5′GGGCCTGTATCCAGCCTCAG3′/5′GAGCCGGTACAGCCAATGGT3′;
IFN‐λ3, 5′GGGCCTGTATCCAGCCTCAG3′/5′GGTGCAGCCAATGGTGGAG3′/;
LL‐37, 5′TTTTGCGGAATCTTGTACCCA3′/5′TCTCAGAGCCCAGAAGCCTG3′;
GAPDH, 5′TGGACCTGACCTGCCGTCTA3′/5′CCCTGTTGCTGTAGCCAAATTC3′.

Samples were analyzed using the QuantStudio5 Real‐Time PCR System (Thermo‐Fisher Scientific, Waltham, MA, USA).

### SNP analysis

DNA was extracted from blood using the QIAcube® system (Qiagen, Hilden, Germany). SNPs were selected based on an extensive review of articles on the association between psoriasis and SNPs or response to biologics 23, 24, 28, 29, 30, 31, 32. The SNP panel was analyzed by targeted sequencing, using NGS TruSeq Custom Amplicon kit and the MiSeq platform (Illumina, San Diego, CA, USA). SNPs are listed in supplementary material, Table S1 together with additional SNPs near the investigated genomic regions. Positive calls were selected applying a read depth >50X and allelic frequency >0.3. Variants' annotations were verified with ANNOVAR on hg19.

### Statistics

Wilcoxon's signed rank test (SigmaStat; San Rafael, CA, USA) was used to compare differences in mRNA content in skin biopsies of HS and psoriatic patients. The significance of differences in the numbers of immunoreactive cells in skin biopsies was calculated using the unpaired Student's *t*‐test. Statistical analysis was performed with Prism v.5.0 (Graphpad, La Jolla, CA, USA), and values are expressed as the mean + SD. Values of *p* < 0.05 were considered significant.

## Results

### Clinical characterization of paradoxical psoriasis

**Figure 1:**
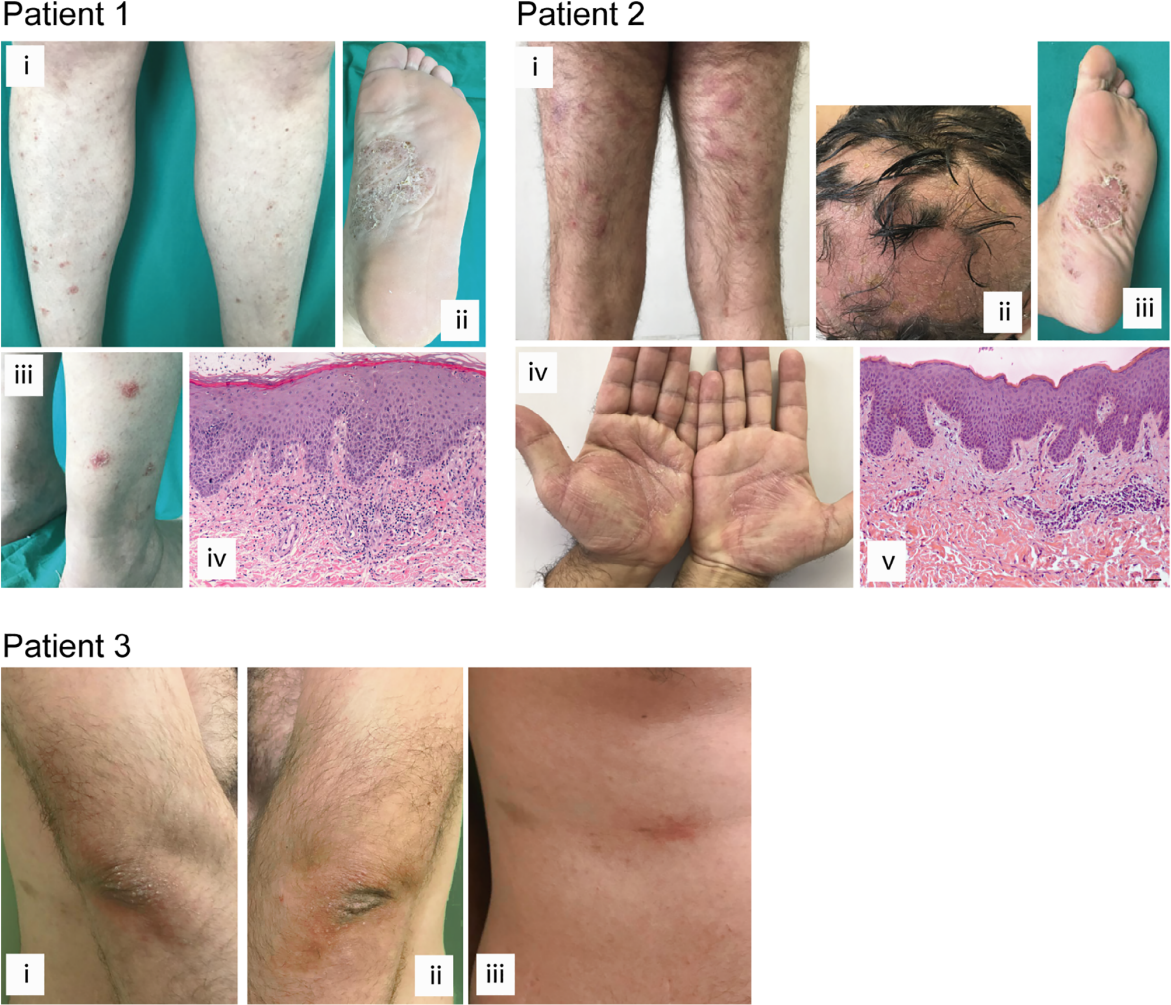
Clinical and histological presentation of paradoxical psoriasis induced by anti‐TNF‐α therapy in HS patients. Cutaneous lesions of patients 1–3 affected by severe HS, presenting paradoxical psoriasiform reaction after anti‐TNF‐α treatment. The patient 1 panels show paradoxical erythemato‐squamous plaques localized on the lower limbs (i and iii) and pustular lesions in the plantar region (ii). Patient 2 similarly shows erythemato‐squamous plaques on the limbs (i), pustular lesions in the palmo‐plantar region and a severe form of alopecia areata involving part of the scalp (ii–iv).The patient 3 panels reveal erythematosus patches with mild desquamation on the elbows and trunk (i–iii). H&E staining for the corresponding histopathology of patients 1 (iv) and patient 2 (v) was also performed. Scale bars, 200 μm.

We analyzed three patients affected by severe HS, and who developed paradoxical psoriasiform reactions following treatment with adalimumab. Patient 1, a 48‐year‐old Caucasian woman, showed nodules, fistulas and sinus tracts in the inguinal and perianal regions (Hurley III, Sartorius score: 41.5). After 3 months of therapy with anti‐TNF‐α, she developed psoriasiform eruptions (PASI 6.8), with pustular lesions and erythemato‐scaly lesions on the plantar region and lower limbs, respectively (Figure 1). A similar pattern of HS severity was observed in patient 2 (Hurley III, Sartorius score: 41.5), a 40‐year‐old Caucasian man, showing erythematous‐pustular lesions in the palmo‐plantar regions and erythemato‐scaly plaques on the legs and scalp, ascribed to psoriasiform dermatitis (PASI 5.2), arising after 2 months treatment with adalimumab (Figure 1). He concomitantly showed alopecia areata on the scalp and some eczematous‐like skin lesions. Patient 3, a 27‐year‐old Caucasian man, was affected by severe HS (Hurley III, Sartorius score: 61.5) characterized by comedones, nodules, and fistulas in the inguinal, gluteal and abdominal regions. He developed pustular lesions in the palmo‐plantar regions, and erythemato‐scaly plaques on the legs, scalp, elbows, and trunk (PASI 5.6) after 3 months of biological therapy (Figure 1). He refused to undergo a punch biopsy and, therefore, we could not perform the histological and immunological analyses. Paradoxical psoriasis regressed in all patients when adalimumab was discontinued. Interestingly, all three patients examined had a positive family history for psoriasis and, additionally, patient 1 reported other cases of HS among first‐degree relatives.

**Figure 2:**
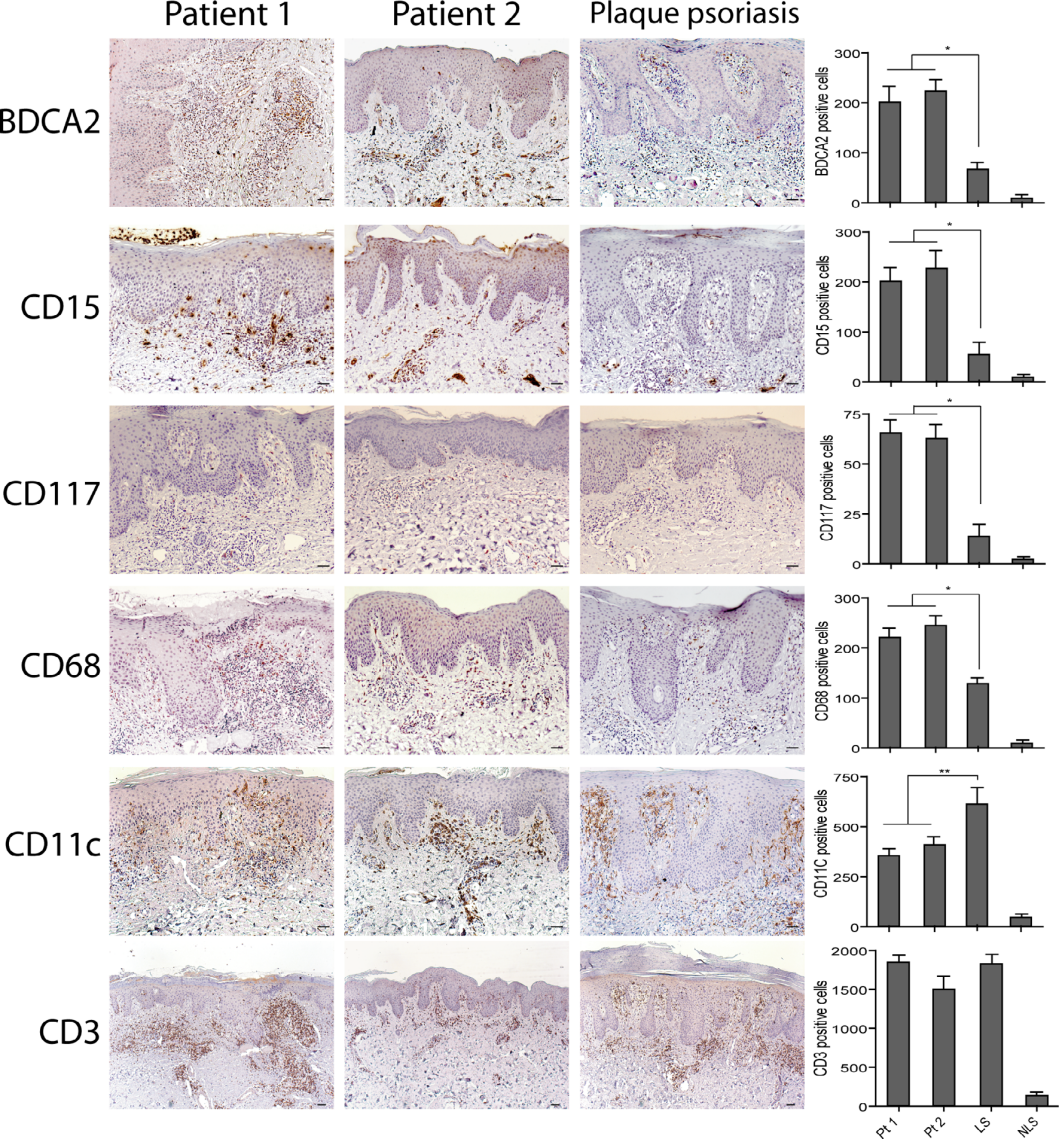
Innate immunity cells highly infiltrate paradoxical psoriasis skin lesions. Immunohistochemistry analysis of paradoxical skin reactions obtained from patient 1 (Pt1) and 2 (Pt2) shows an increase of BDCA2, CD15, CD117, CD68 positive cells, a reduction of CD11c cells and similar numbers of CD3 cells, when compared with psoriasis. Lesional (LS) and nonlesional (NLS) skin of the same psoriatic patient (*n* = 3) was analyzed. Slides were analyzed by two pathologists with experience in dermatology. Positive cells were counted in five adjacent fields at a total magnification of ×200. Graphs show the mean number of positive cells + SD *per* three sections. One out of three representative stainings is shown. *p < 0.01, **p < 0.05 versus classical psoriasis. Scale bars, 200 μm.

Histological examination of the psoriasiform lesions of patients 1 and 2 showed epidermal hyperplasia with parakeratosis, papillary vessel ectasia and perivascular infiltrate compatible with a psoriasiform dermatitis (Figure 1). A CD15^+^ neutrophilic infiltrate was abundant in the dermal compartment and present in corneal abscesses (Figures 1 and 2). Interestingly, some eczematiform spongiotic areas overlapping with the psoriasis‐like histological pattern were present in the skin lesions of patient 2.

### Innate immunity cells highly infiltrate paradoxical psoriasiform lesions

Leukocyte subpopulations were characterized in paradoxical psoriasiform lesions, and compared to those present in classical plaque‐type psoriasis. In line with previous studies 19, 33, paradoxical psoriasis exhibited a prominent infiltrate of BDCA2^+^ pDCs in the dermis, significantly more abundant than in classical psoriasis (~2.7‐fold increase). In parallel, a significant increase of CD15^+^ neutrophils, c‐kit/CD117^+^ mast cells, CD68^+^ macrophages and monocytes in the dermis of paradoxical skin reactions was observed (~3.8‐, 3.5‐, and 1.8‐fold increase, respectively) (Figure 2). In contrast, CD3^+^ cells were similar and CD11c^+^ DCs were less abundant (~1.5 fold‐decrease) (Figure 2).

**Figure 3:**
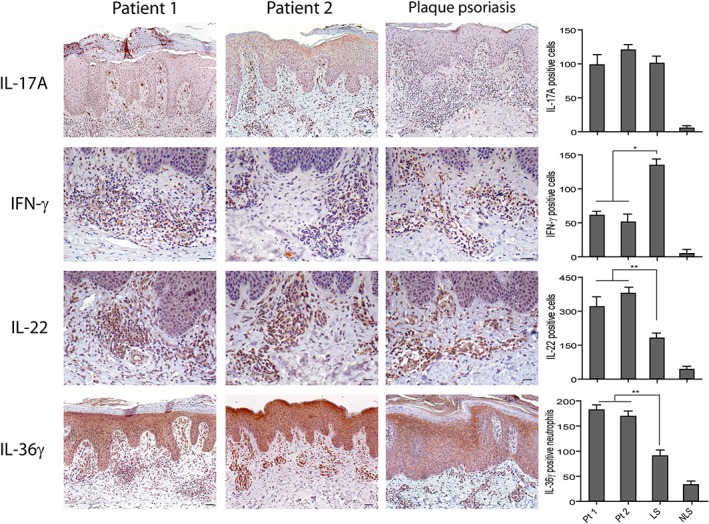
Expression of psoriasis‐related cytokines in paradoxical psoriasiform reactions. Immunohistochemistry analysis performed on paradoxical skin lesions obtained from patients 1 (Pt1) and 2 (Pt2) shows similar values of IL‐17A^+^ cells, a reduction of dermal IFN‐γ^+^ cells and an increase of IL‐22^+^ or IL‐36γ^+^ cells, when compared with psoriatic skin lesions. LS and NLS skin of the same psoriatic patient (*n* = 3) was analyzed. Graphs show the mean of number of positive cells + SD *per* three sections. One out of three representative stainings is shown. *p < 0.01, **p < 0.05, versus classical psoriasis. Scale bars, 200 μm.

**Figure 4:**
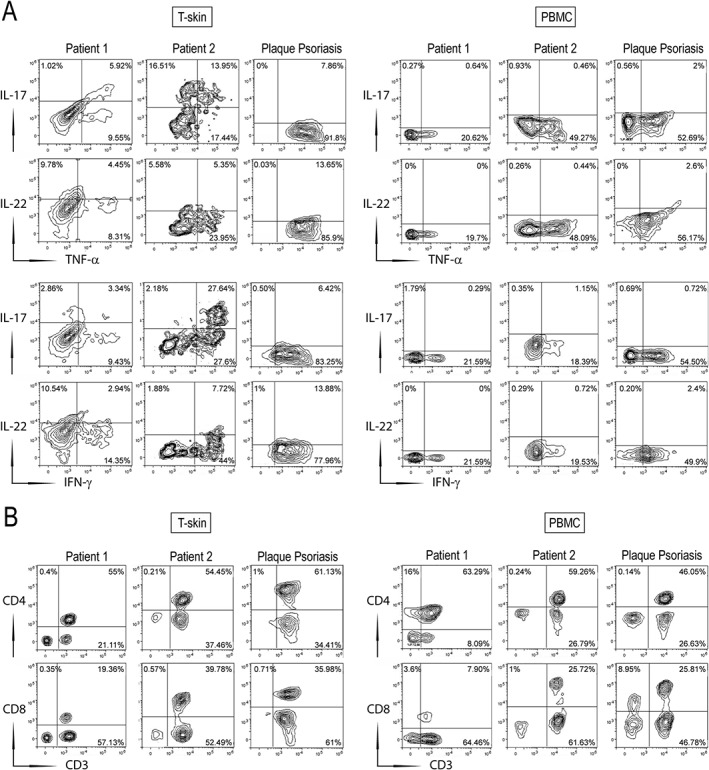
Immunophenotypic characterization of skin T cells (T‐skin) and PBMCs isolated from HS patients with psoriasiform lesions. T‐skin cells (left panel) and PBMCs (right panel) were isolated from biopsies and blood, respectively, of patients 1 and 2 and from psoriatic patients (*n* = 2). Co‐expression of IL‐17, IL‐22, TNF‐α, or IFN‐γ on gated CD3^+^ cells (A), and surface CD4, CD8, and CD3 (B), were analyzed by flow cytometry. The percentage of positive fluorescent cells is shown in each quadrant. The results show the mean values of data obtained for one representative experiment out of three experiments.

We next evaluated the local expression of psoriasis‐related cytokines, such as IL‐17A, IFN‐γ, and IL‐22, as well as IL‐36γ which is highly released by neutrophils 27. As shown in Figure 3, IFN‐γ immunoreactivity decreased in psoriasiform reactions of patients 1 and 2, as compared to classical psoriasis, whereas IL‐22 positivity was significantly augmented in the infiltrate, in particular in cells with a macrophage‐like morphology (~2.1‐fold increase). Due to the numerous neutrophils present in the dermis of paradoxical reactions, IL‐36γ positivity was also enhanced, compared to classical psoriasis. However, IL‐36γ expression in the epidermal compartment was similar (Figure 3). FACS analysis of the T‐cells isolated from skin biopsies confirmed a significant reduction of IFN‐γ^+^ CD3^+^ cells in patients 1 and 2, when compared to CD3^+^ cells isolated from classical psoriasis (~7‐ and 1.7‐fold decrease, respectively) (Figure 4). The reduction of IFN‐γ positivity was also observed in circulating CD3^+^ cells of patients 1 and 2. Similarly, TNF‐α positivity was lower in skin T‐lymphocytes of patients 1 and 2. TNF‐α positivity of circulating CD3^+^ cells was instead lower only in patient 1, as compared to patient 2 and patients with classical psoriasis (Figure 4). Moreover, IL‐17A positivity was comparable in patient 1 and psoriatic patients, whereas it was very high in T cells isolated from the skin of patient 2, where a mixed population of T cells, responsible for either the psoriasiform or eczematous reactions, is likely present (Figure 1). IL‐22 was similar in T cells of psoriasiform lesions and classical psoriasis, whereas it was substantially reduced in PBMCs (Figure 4). CD3^+^ cells from skin biopsies of HS patients were enriched in CD8^+^, but not in CD4^+^ cells, when compared to PBMC isolated from the same patients. CD3^+^ skin T cells of classical psoriasis showed instead an enrichment of both CD4^+^ and CD8^+^ subpopulations (Figure 4).

### Overexpression of innate immunity molecules in paradoxical psoriasis

**Figure 5:**
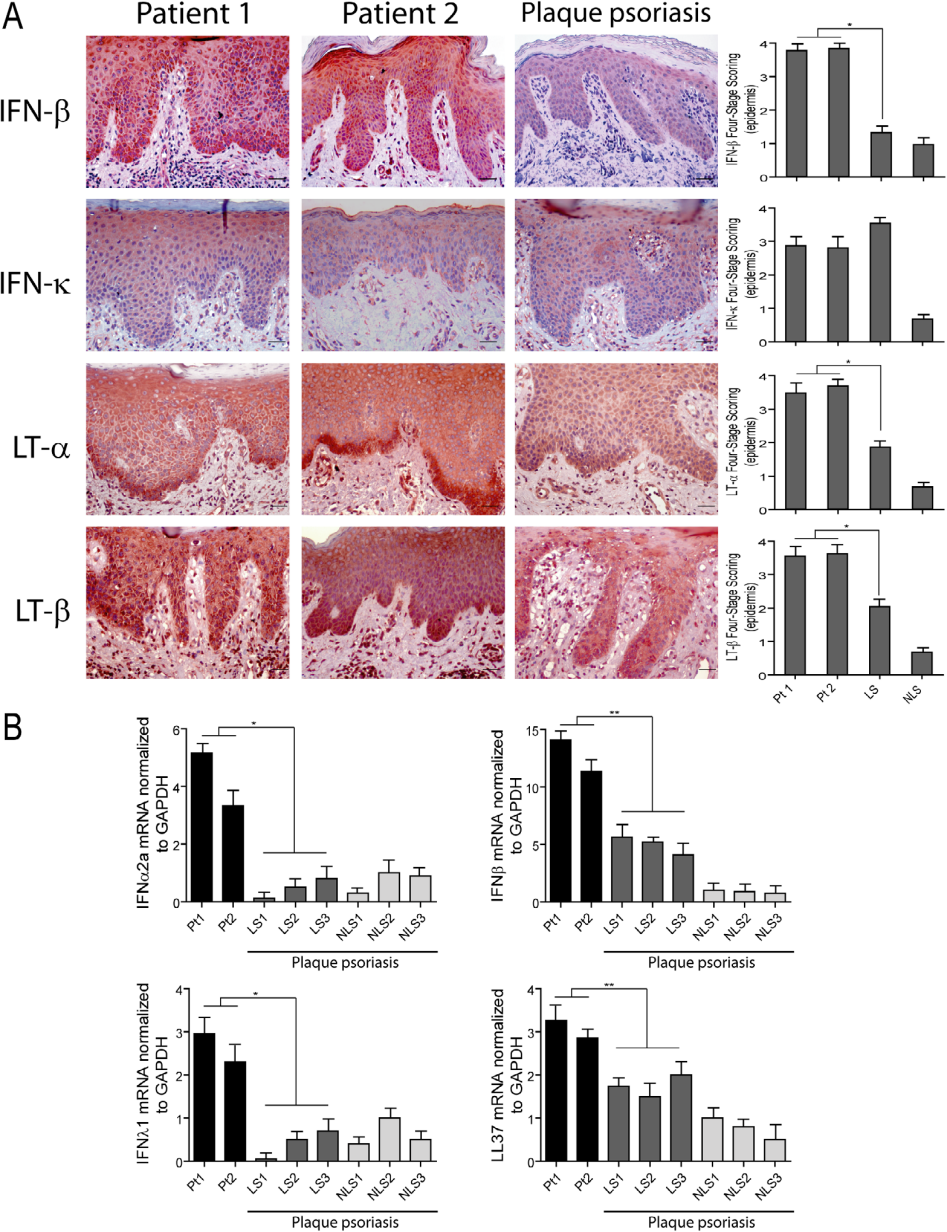
Innate immunity molecules are overexpressed in the skin of HS patients after TNF‐α treatment. (A) Immunohistochemistry analysis of paradoxical skin reactions obtained from patients 1 (Pt1) and 2 (Pt2) shows an increase of IFN‐β, LT‐α, LT‐β, and similar IFN‐κ positivity, when compared with psoriatic skin lesions. LS and NLS skin of the same psoriatic patient (*n* = 3) was analyzed. Graphs show the mean + SD of semiquantitative, four‐stage scoring, ranging from negative immunoreactivity (0) to strong immunoreactivity (4+) and relative to the epidermal expression of the indicated molecules. One out of three representative stainings is shown. *p < 0.01, versus classical psoriasis. Scale bars, 200 μm. (B) mRNA expression of *IFNα2a*, *IFNβ*, *IFNλ1* and *LL37* was analyzed by real‐time PCR in skin lesions of patients 1 (Pt1) and 2 (Pt2) and in skin biopsies from LS and NLS skin of three psoriatic patients. mRNA values were normalized to *GAPDH* mRNA. Values obtained from triplicate experiments were averaged, and data presented as means of 2^‐ΔΔCT + SD. *p < 0.01, **p < 0.05.

Since we found that the inflammatory infiltrate pattern in paradoxical psoriasis strongly resembles that present in acute psoriasis, we next analyzed selected innate immunity molecules potentially involved in the triggering of psoriasis. As shown in Figure 5A, the type I IFN‐β was expressed in paradoxical skin lesions, mainly in keratinocytes, at levels significantly higher than classical psoriasis (~1.9‐fold increase). IFN‐β expression was also detected in cells with a T‐cell‐ and DC‐like morphology, as well as in endothelial cells. The epidermis of psoriasiform reactions was also immunoreactive for IFN‐κ, another keratinocyte‐derived type I IFN 34. IFN‐κ expression was similar in the two psoriasis conditions, even if it showed different subcellular localization within keratinocytes, being cytoplasmic in psoriasiform lesions and membrane‐bound in classical psoriasis (Figure 5A). IFN‐κ staining was also present in cells with a monocyte‐ or DC‐like morphology, at comparable levels in classical and nonclassical psoriasis (Figure 5A). Similarly to IFN‐β, IFN‐α2a and IFN‐λ1, but not IFN‐λ2 and IFN‐λ3, were greatly increased in paradoxical psoriasis, as compared to plaque psoriasis (Figure 5B and data not shown). Of note, LL‐37 was strongly expressed in psoriasiform skin lesions, at levels higher than classical psoriasis (Figure 5B). We finally investigated LT‐α and LT‐β, two members of the cytokine TNF family, also known as TNF‐β and TNF‐C 35, possibly deregulated by anti‐TNF‐α therapy. Both lymphotoxins were strongly overexpressed in paradoxical skin reactions, especially in keratinocytes of the basal layer epidermis.

As a whole, these data reveal the presence of an overexpressed innate immunity pattern in the skin of HS patients with paradoxical psoriasis.

### SNP characterization in HS patients developing paradoxical psoriasis

In order to understand whether paradoxical reactions of HS patients had a genetic basis, we analyzed a number of SNPs predisposing to psoriasis. Among them, we studied SNPs frequent in the psoriatic population, such as polymorphisms of the *HLA‐C* and *HLA‐B* regions and the *ERAP1* gene. We also analyzed genetic variants of pathogenic cytokines, receptors and signal transducers (i.e. TNF‐α, IL‐17F, IL‐17RA, IL‐23R, IL‐12B, IKBζ, A20, A20 binding protein, Tyk2), and of skin‐barrier proteins (i.e. CDSN, CCHCR1) (see supplementary material, Table S1). All HS patients showed variants of *ERAP1* and the *HLA‐C* region, either homozygosis or heterozygosis (Table 1). Three SNPs in *ERAP1* (rs30187/rs30186/rs26653) and nine variants in *HLA‐C* (rs114395371/rs9264942/rs10484554/rs2524095/rs28383849/rs9264944/rs2853922/rs147538049/rs9264946) were, in fact, found in all patients, with SNPs distributed differently in the three patients. None of them showed the classical *HLA‐Cw6* allele, even though patients 1 and 3 carried three point SNPs (rs2524095/rs2853922/rs386698994) mapping near the *HLA‐Cw6* SNP position (rs17192540) (Table 1). In contrast, patient 2 mostly showed genetic polymorphisms (rs9264942/rs10484554/rs28383849/rs9264944/rs147538049/rs9264946) present in the genomic region containing a second variant of *HLA‐Cw6* (Table 1). All HS patients carried SNPs in *NFKBIZ* (rs3217713) and *TNFAIP* (rs610604) genes, encoding IKBζ and A20 proteins respectively. Interestingly, patient 3 showed the higher number of psoriasis‐related SNPs, and shared a number of SNPs with patient 2 (rs7637230/rs4819554/rs3132554/rs10542126/rs3130983), and rs280519 with patient 1 (Table 1). Patient 3 also carried two other SNPs in *CDSN* (rs1062470/rs707913) and three SNPs in *CCHCR1* (rs1576/rs130079/rs746647) (Table 1).

**Table 1:** SNPs carried by HS patients developing paradoxical psoriasis after anti‐TNF‐α therapy

dbSNP ID	Gene	Patient 1	Patient 2	Patient 3
Antigen presentation				
rs30187	ERAP1	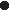	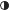	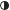
rs30186	ERAP1	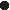	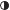	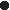
rs11743410	ERAP1	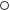	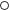	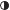
rs26653	ERAP1	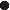	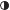	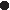
rs114395371	HLA‐C region	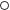	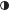	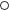
rs17192540	HLA‐C region (HLA‐Cw6)	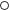	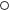	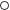
rs2524095	HLA‐C region	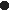	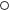	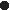
rs2853922	HLA‐C region	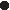	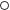	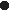
rs386698994	HLA‐C region	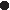	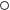	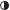
rs79709508	HLA‐C region (HLA‐Cw6 2v)	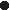	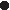	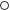
rs28383849	HLA‐C region	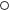	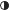	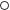
rs10484554	HLA‐C region	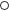	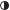	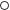
rs147538049	HLA‐C region	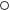	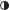	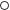
rs9264944	HLA‐C region	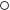	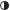	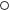
rs9264946	HLA‐C region	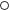	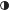	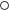
NF‐κB pathway and T‐cell activation				
rs72676067	IL23R	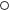	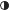	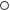
rs1004819	IL23R	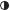	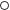	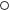
rs41313262	IL23R	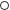	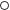	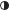
rs11209026	IL23R	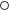	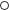	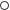
rs3217713	NFKBIZ	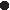	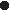	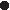
rs7637230	NFKBIZ	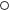	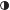	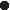
rs2546890	IL12B	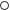	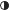	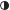
rs1800610	TNF‐α	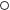	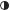	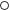
rs2397084	IL17F	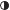	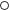	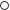
rs71562288	TRAF3IP2	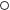	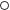	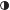
rs33980500	TRAF3IP2	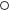	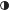	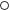
rs610604	TNFAIP3	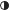	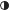	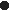
rs12720356	TYK2	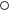	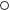	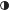
rs280519	TYK2	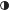	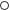	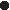
rs4819554	IL17RA	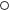	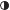	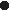
Skin barrier function				
rs3132554	CDSN	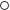	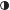	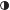
rs1042127	CDSN	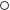	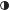	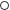
rs1042126	CDSN	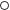	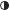	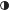
rs1062470	CDSN	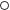	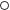	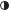
rs707913	CDSN	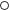	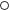	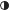
rs3130983	CDSN	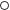	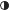	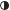
rs1576	CCHCR1	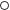	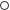	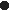
rs130079	CCHCR1	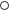	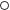	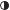
rs746647	CCHCR1	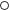	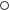	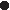
rs130075	CCHCR1	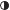	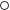	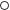

DNA was obtained from blood samples of HS patients 1–3, and SNPs analyzed by next‐generation sequencing technology in MiSeq system, as described in ‘Material and methods’ section. For each sample, a cDNA library of 44 amplicons potentially containing 71 SNPs located in genes predisposing to psoriasis was developed. SNP‐carrying genes were classified accordingly to their functions (i.e. control of antigen presentation, NF‐κB pathway and T‐cell activation, skin barrier).

dbSNP ID, data base SNP identification number at NCBI; rs, reference *SNP* ID number; ERAP1, endoplasmic reticulum aminopeptidase 1; NFKBIZ, NF‐κB inhibitor zeta; TRAF3IP2, TRAF3 interacting protein 2; TNFAIP3, TNF‐α induced protein 3; TYK2, tyrosine kinase 2; IL17RA, IL‐17 receptor A; CDSN, corneodesmosin; CCHCR1, coiled‐coil alpha‐helical rod protein 1. 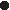: homozygotic variant; 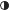: heterozygotic variant; 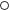: WT. Rs17192540 and rs79709508 were relative to HLA‐Cw6 and HLA‐Cw6 second allelic variant (HLA‐Cw6 2v).

Although rs11209026 in the *IL23R* gene has been previously associated with paradoxical psoriasiform reactions to anti‐TNFs 36, we could not find this SNP in any of the HS patients. Two other SNPs in *IL23R*, rs72676067, and rs1004819, were instead detected in patients 2 and 1, respectively.

## Discussion

Psoriasis pathogenesis involves both innate and adaptive immunity responses, overactive in different clinical phases and characterized by specific patterns of inflammation. While innate immunity processes predominate in early/acute phase, with immune cells such as pDC, neutrophils, mast cells and macrophages being abundant in skin lesions, adaptive immune responses are typical of chronic psoriasis 10, 14, 15, 37. Local overproduction of IFN‐α and other innate immune mediators, such as antimicrobial peptides, also characterize early psoriasis 37, 38. Conversely, during the development of chronicity, type I IFNs are no longer produced, in part due to the inhibitory effects of TNF‐α, which determines the decline of innate immunity processes and the mounting of adaptive immune responses 39. During this phase, TNF‐α is important for immune activation of mDC, which, after encountering the causative antigen(s), are responsible for T‐cell expansion.

In this study, we found that paradoxical psoriasis evoked by anti‐TNF‐α therapy in patients affected by HS strongly resembles early psoriasis. In fact, by comparing skin lesions of paradoxical psoriasis with classical psoriasis, we observed a marked dermal accumulation of innate immunity cells, including pDCs, neutrophils, mast cells, and macrophages. In parallel, the expression levels of innate immunity molecules potentially involved in induction of the psoriasiform phenotype, greatly increased in paradoxical reactions. Among them, IFN‐α2a, IFN‐β, and IFN‐λ1 are overexpressed in the skin of HS patients following anti‐TNF‐α therapy. Also, LT‐α and LT‐β, as well as LL‐37, were detected at very high levels in paradoxical psoriasis, when compared to classical psoriasis. A transient IFN‐α upregulation has already been described in classical psoriasis, during the early phase of disease development, as well as in paradoxical psoriasis 19, 37. Concomitantly, IFN‐β is known to be expressed by pDCs in both conditions 19. We found that IFN‐β, together with IFN‐κ and lymphotoxins were impressively expressed in the epidermal compartment of paradoxical skin reaction, as well as in pDCs and leukocytes infiltrating the dermis. Type I IFNs and lymphotoxins released by keratinocytes might have a fundamental pathogenic role in paradoxical psoriasis. However, the mechanisms by which these molecules promote a psoriatic skin phenotype are not yet known, neither in paradoxical nor in classical psoriasis. On the contrary, the immunological function of IFN‐α has been extensively studied, especially in classical psoriasis, where it is known to induce Th17 responses 40. In paradoxical psoriasis, IFN‐α could have a different role, with antigen‐specific Th17 responses being absent. It could induce chemokines at the epidermal level, such as CXCL10 and CXCL9, responsible for the recruitment of DC and nonspecific T cells. These inflammatory cells could in turn sustain and amplify local inflammatory responses in paradoxical reactions 41.

The induction of innate immunity players in paradoxical psoriasis is dependent on the loss of TNF‐α function in limiting the innate immune responses in the skin, as previously demonstrated 19. In fact, TNF‐α blockade determined the accumulation of pDCs and inhibition of their maturation. As a consequence, pDCs could release very high levels of the type I IFN‐α6 and IFN‐β, being, thus, responsible for paradoxical psoriasis. Together with pDCs, we found other innate immunity cells present in psoriasiform lesions of HS patients. Among them, CD15^+^ neutrophils, c‐kit/CD117^+^ mast cells, CD68^+^ macrophages and monocytes abundantly infiltrate the dermis of paradoxical skin reactions. This pattern of leukocyte subpopulations is very similar to that found in early psoriasis, and is consistent with the high local production of IL‐36 cytokines and with the overactive innate immunity processes present during the initial phase of psoriasis, as previously shown 14, 27. Conversely, similarly to paradoxical psoriasis, adverse HS in patients affected by autoimmune disorders, including psoriasis and Crohn's disease, might be dependent on aberrant innate immunity responses evoked by TNF‐α blockade 42, 43. Indeed, a number of pathogenic cytokines common to psoriasis were found in HS skin, including IL‐36, together with inflammatory mediators active on neutrophils and Th17 cells largely present in the affected areas 27. Other than pDCs, innate immune cells could also be recruited by chemokines released by keratinocytes, fibroblasts and endothelial cells (i.e. CCL20, chemerin), whose expression depends on type I IFNs produced by resident skin cells themselves. In fact, other than controlling the expression of type I IFNs in pDCs, TNF‐α might negatively regulate these molecules in keratinocytes, which notoriously also contribute to the induction of innate immunity pathways in early psoriasis 10. This hypothesis is supported by our findings that type I IFNs are induced in keratinocytes of paradoxical psoriasis and vice versa are present at low levels in chronic plaque psoriasis. It would be important to confirm the high expression of innate immunity mediators following TNF‐α blockade *in vitro* in primary keratinocyte cultures, as demonstrated for cultured pDCs. It would be also relevant to analyze whether, similarly to paradoxical psoriasis, acute psoriasis shows exaggerated expression of type I IFNs and lymphotoxins in the epidermal compartment. In that case, it can be supposed that TNF‐α temporally limits innate immunity processes evoked not only by pDCs but also by keratinocytes to unleash adaptive immune responses in psoriatic skin.

Concerning the unknown expression and role of lymphotoxins in paradoxical reactions and in classical psoriasis, a previous study confirmed the pivotal function of LT‐α, together with TNF‐α, in determining NF‐κB‐mediated skin inflammatory reactions in IκBα^−/−^ mice 44. In addition, patients affected by psoriatic arthritis treated with etanercept showed increased serum levels of LT‐α 45. Therefore, lymphotoxins might be deeply involved in psoriasis pathogenesis, and TNF‐α could tightly control their expression in both keratinocytes and lymphocytes. Further studies are needed to evaluate LT‐α and LT‐β expression and their role in the different phases of psoriasis development, and to understand the function of keratinocyte‐derived lymphotoxins.

Analysis of the T‐cell infiltrate in paradoxical skin reactions demonstrated a significant reduction of IFN‐γ‐ or TNF‐α‐producing CD3^+^ cells in paradoxical psoriasis, when compared to chronic psoriasis. However, CD8^+^ and IL‐17^+^ lymphocytes were present in paradoxical psoriasiform reactions, at levels comparable to psoriasis, even if it is conceivable that they were nonspecifically recruited. The absence of bursting of a type I IFN T‐cell response in paradoxical skin reactions was not surprising, if we consider that it is typical of the chronic phase in classical psoriasis 10, 11, 15. On the contrary, IL‐22‐producing cells increased in psoriasiform reactions of HS patients, even though positive cells showed mostly a macrophage‐like morphology. Our findings extend previous studies showing the upregulation of IL‐22 mRNA expression in paradoxical psoriasis, and identifying innate immunity cells, and not only T lymphocytes, as cellular sources of IL‐22 19. IL‐22 overexpression could be responsible for hyperproliferation and de‐differentiation of keratinocytes typical of the epidermis of paradoxical psoriasiform lesions. Finally, although an inflammatory cytokine milieu, inducing the local production of chemokines and cytokines by resident skin cells, can be effectively established in paradoxical psoriasis, it seems to be insufficient to induce the development of chronic psoriasiform reactions in HS patients, possibly through the lack of mDC and T‐cell activation by the causative antigen(s) of psoriasis.

Despite the effective use of adalimumab in patients with severe HS, 2–5% of treated patients develop paradoxical psoriasis 6. Anti‐TNF‐α treatment can induce paradoxical psoriasis even in patients affected by other diseases characterized by high levels of TNF‐α 8, 46, 47. Notably, this side effect can also occur in patients undergoing psoriasis treatment with anti‐TNFs. Guttate or pustular forms in palmo‐plantar/scalp areas frequently represent the subclinical types of psoriasis that develop in these reactive patients. The reason why anti‐TNF induces a similar psoriatic phenotype (same subtype and localizations) only in a portion of subjects affected by different autoimmune conditions is still unknown. It is reasonable to speculate the influence of genetic factors predisposing to paradoxical psoriasis, and specifically being involved in innate immunity pathways, in particular in pDC activation and/or type I IFN and TNF‐α signaling. Indeed, an association between polymorphisms in the *IL‐23R*, *FBXL19*, *CTLA4*, *SLC12A8*, and *TAP1* genes and paradoxical psoriasis has been found 36. On the other hand, there is a positive association between HS and psoriasis, with the prevalence of HS increased in patients with psoriasis, suggesting a common genetic predisposition 48. To date, no evidence correlating the presence of SNPs and the development of psoriasiform lesions in patients affected by HS exist. In our study, we found that all HS patients carried numerous allelic variants in *HLA‐C*. None of the patients showed the *HLA‐Cw6* susceptibility allele, even though other SNPs in the proximity of the *HLA‐Cw6* SNP and neighboring to other *HLA‐C* variants were found. Concomitantly, HS patients carried allelic variants in the *ERAP1* gene. However, due to the lack of antigen‐specific CD8+ T‐cell responses in HS patients, the link between the presence of SNPs in the *HLA‐C* region/*ERAP1* gene and susceptibility to paradoxical psoriasis is apparently missing. Indeed, other than having a role in MHC class I antigen presentation, ERAP1 is involved in the activation of innate immunity pathways, by inducing the inflammasome and production of cytokines and chemokines (i.e. IL‐6, TNF‐α, and CCL2) 49. Importantly, allelic variants of *ERAP1* leading to missense mutation increase the capability of ERAP1 to induce inflammation in autoimmune diseases 50. HS patients also carried polymorphisms in *NFKBIZ* and *TNFAIP3*, which could be responsible for NF‐κB hyperactivation in HS patients, as demonstrated for other pathological conditions 51, 52. Importantly, allelic variants in *NFKBIZ* and *TNFAIP3* might determine the enhanced type I IFN expression observed in pDCs and keratinocytes of paradoxical lesions, as both IKBζ and A20 can transcriptionally regulate IFN expression, respectively, via activation and inhibition of NF‐κB 53. Finally, genetic variants in *TNFAIP3*, in particular those imparting lower A20 expression, might be responsible for uncontrolled IFN‐β expression, as demonstrated by silencing *TNFAIP3* mRNA expression in a vascular model of inflammation 54. All these SNPs in psoriasis susceptibility loci are likely genetically transmitted, as all three patients examined had a positive family history for psoriasis. In the future, it will be necessary to extend the analysis of psoriasis‐related SNPs to a larger cohort of HS patients developing psoriasiform reactions, but also in a population successfully responding to anti‐TNF‐α treatment, to identify differences in the genetic background of the patients. The identification of genetic biomarkers correlating with an adverse response to anti‐TNF‐α therapy will be fundamental to predict the risk of developing paradoxical psoriasis.

In conclusion, our study shows that paradoxical psoriasis induced by anti‐TNF in patients affected by HS has immunological features common to early phase psoriasis, mainly characterized by cellular and molecular players of innate immunity. Among them, LT‐α and LT‐β, as well as IFN‐κ and IFN‐λ1, have been identified as new innate mediators potentially involved in the induction of paradoxical psoriasis. Of note, we found that, in addition to pDCs, keratinocytes are also a source of type I IFNs, in particular IFN‐β, likely as consequence of TNF‐α inhibition. It will be important to evaluate the effects of anti‐TNF‐α therapy on keratinocytes in paradoxical psoriasiform reactions, especially in terms of type I IFN production, to identify new pathogenic mechanisms involved in the early phase of psoriasis.

## Author contributions statement

LF, TT, MAP, GP, and CM enrolled HS and psoriatic patients, and collected clinical data. MM, CS, LM, and SM performed immunohistochemical studies, RNA and genetic analyses. GLS performed bioinformatics analysis of SNPs. FS and CC isolated and analyzed T cells of skin biopsies and blood of HS patients. LF, MM, SM, and CA designed and interpreted the experiments. CA wrote the manuscript, and, together with MM, compiled the figures. LF, MM, GG, AC, and SM made revisions and proofread the manuscript.

## Supporting information


**Table S1.** List of the analyzed SNPs
