# Supplementary material for: Paradoxical psoriasis induced by TNF‐α blockade shows immunological features typical of the early phase of psoriasis development
Source: J Pathol Clin Res. 2019 Oct 29;6(1):55–68. doi: 10.1002/cjp2.147 (PMC6966707; doi:10.1002/cjp2.147)
Supplement: Supplementary file 1 — Table S1. List of the analyzed SNPs [file CJP2-6-55-s001.docx]

**Paradoxical psoriasis induced by TNF-α blockade shows immunological features typical of the early phase of psoriasis development**

Fani L *et al*. *J Pathol Clin Res* DOI: 10.1002/cjp2.147

**Table S1.** List of the analyzed SNPs

| Antigen presentation | | | | | | | | | | | | | |  |
| --- | --- | --- | --- | --- | --- | --- | --- | --- | --- | --- | --- | --- | --- | --- |
| dbSNP ID | | | **Genome position** | | | | | **Gene** | **REF>ALT** | | | | |  |
| rs27524 | | | Chr5:96101944 | | | | | ERAP1 | A>G | | | | |  |
| rs27525* | | | Chr5:96101959 | | | | | ERAP1 | C>T | | | | |  |
| rs30187 | | | Chr5:96124330 | | | | | ERAP1 | T>C | | | | |  |
| rs30186* | | | Chr5:96124447 | | | | | ERAP1 | G>C | | | | |  |
| rs11743410* | | | Chr5:96124453 | | | | | ERAP1 | A>G | | | | |  |
| rs26653 | | | Chr5:96139250 | | | | | ERAP1 | C>G | | | | |  |
| rs114395371 | | | Chr6:31252925 | | | | | HLA-C region | C>T | | | | |  |
| rs116350468 | | | Chr6:31252951 | | | | | HLA-C region | G>T | | | | |  |
| rs115727572 | | | Chr6:31253034 | | | | | HLA-C region | T>G | | | | |  |
| rs17192533 | | | Chr6:31266085 | | | | | HLA-C region | C>G | | | | |  |
| rs17192540 | | | Chr6:31266090 | | | | | HLA-C region | G>A | | | | |  |
| rs2524095 | | | Chr6:31266117 | | | | | HLA-C region | A>C | | | | |  |
| rs7761855 | | | Chr6:31266151 | | | | | HLA-C region | G>T | | | | |  |
| rs386698993 | | | Chr6:31266189 | | | | | HLA-C region | AA>TG,AG | | | | |  |
| rs2853922 | | | Chr6:31266190 | | | | | HLA-C region | A>G | | | | |  |
| rs386698994 | | | Chr6:31266207 | | | | | HLA-C region | CA>TG | | | | |  |
| rs79709508 | | | Chr6:31274380 | | | | | HLA-C region | T>C | | | | |  |
| rs35647108 | | | Chr6:31274449 | | | | | HLA-C region | C>A | | | | |  |
| rs6931873 | | | Chr6:31274513 | | | | | HLA-C region | A>G | | | | |  |
| rs28383849 | | | Chr6:31274518 | | | | | HLA-C region | T>TCGGGGAGTCCAGCAGGTCC,TCCGGGAGTCCAGCAGGTCC | | | | |  |
| rs10484554 | | | Chr6:31274555 | | | | | HLA-C region | C>T | | | | |  |
| rs184149624 | | | Chr6:31274580 | | | | | HLA-C region | CAGCCAA>GAGCCAA,CAACCAG | | | | |  |
| rs9348865 | | | Chr6:31274582 | | | | | HLA-C region | GCCAA>ACCAG,GCCAG | | | | |  |
| rs147538049 | | | Chr6:31274584 | | | | | HLA-C region | CA>C | | | | |  |
| rs9348865 | | | Chr6:31274586 | | | | | HLA-C region | A>G | | | | |  |
| rs9264944 | | | Chr6:31274619 | | | | | HLA-C region | A>G | | | | |  |
| rs9264946 | | | Chr6:31274634 | | | | | HLA-C region | T>C | | | | |  |
| rs2395029 | | | Chr6:31431780 | | | | | HLA-B region | T>G | | | | |  |
| rs2243621* | | | Chr6:31431820 | | | | | HLA-B region | C>T | | | | |  |
| rs2395030* | | | Chr6:31431874 | | | | | HLA-B region | G>T | | | | |  |
| NF-kB pathway and T-cell activation | | | | | | | | | | | | | | |
| dbSNP ID | | **Genome position** | | | | **Gene** | | | | **REF>ALT** | | | | |
| rs72676067 | | | | Chr1:67658803 | | | IL23R | | | | | G>A | | |
| rs1004819 | | | | Chr1:67670213 | | | IL23R | | | | | G>A | | |
| rs41313262* | | | | Chr1:67705900 | | | IL23R | | | | | G>A | | |
| rs11209026 | | | | Chr1:67705958 | | | IL23R | | | | | G>A | | |
| rs144478519 | | | | Chr2:113820124 | | | IL1F5 | | | | | C>T | | |
| rs3217713 | | | | Chr3:101576029 | | | NFKBIZ | | | | | T>TACTTTTAGAAAGCTTTAATAACC | | |
| rs7637230 | | | | Chr3:101663555 | | | NFKBIZ | | | | | A>G | | |
| rs2233278 | | | | Chr5:150467189 | | | TNIP1 | | | | | G>C | | |
| rs3212227 | | | | Chr5:158742950 | | | IL12B | | | | | T>G | | |
| rs2546890 | | | | Chr5:158759900 | | | IL12B | | | | | A>G | | |
| rs1800629 | | | | Chr6:31543031 | | | TNFα | | | | | G>A | | |
| rs361525 | | | | Chr6:31543101 | | | TNFα | | | | | G>A | | |
| rs1800610 | | | | Chr6:31543827 | | | TNFα | | | | | G>A | | |
| rs374501689* | | | | Chr6:31543943 | | | TNFα | | | | | G>GTGAA | | |
| rs56499381 | | | | Chr6:52101739 | | | IL17F | | | | | T>C | | |
| rs11465553* | | | | Chr6:52101758 | | | IL17F | | | | | C>T | | |
| rs2397084 | | | | Chr6:52101844 | | | IL17F | | | | | T>C | | |
| rs71562288 | | | | Chr6:111577761 | | | Act1 | | | | | A>G | | |
| rs33980500 | | | | Chr6:111913262 | | | Act1 | | | | | C>T | | |
| rs13210247 | | | | Chr6:111922720 | | | Act1 | | | | | | A>G | |
| rs2230926 | | | | Chr6:138196066 | | | TNFAIP3 | | | | | | T>G | |
| rs610604 | | | | Chr6:138199417 | | | TNFAIP3 | | | | | | G>T | |
| rs146214639* | | | | Chr17:78157811 | | | CARD14 | | | | | | T>G | |
| rs12720356 | | | | Chr19:10469975 | | | TYK2 | | | | | | A>C | |
| rs280519 | | | | Chr19:10472933 | | | TYK2 | | A>G | | | | | |
| rs4819554 | Chr22:17565035 | | | | IL17RA | | | | | | G>A | | | |
| Skin barrier function | | | | | | | | | | | | | | |
| dbSNP ID | **Genome position** | | | | **Gene** | | | | | | **REF>ALT** | | | |
| rs3132554 | Chr6:31084163 | | | | CDSN | | | | | | A>G | | | |
| rs1042127 | Chr6:31084170 | | | | CDSN | | | | | | A>C | | | |
| rs33941312 | Chr6:31084191 | | | | CDSN | | | | | | T>C | | | |
| rs1042126* | Chr6:31084288 | | | | CDSN | | | | | | T>C | | | |
| rs1062470 | Chr6:31084435 | | | | CDSN | | | | | | G>A | | | |
| rs707913 | Chr6:31084787 | | | | CDSN | | | | | | A>G | | | |
| rs3130983* | Chr6:31084792 | | | | CDSN | | | | | | C>T | | | |
| rs1576 | Chr6:31110391 | | | | CCHCR1 | | | | | | G>C | | | |
| rs130079 | Chr6:31112737 | | | | CCHCR1 | | | | | | C>A | | | |
| rs746647 | Chr6:31114182 | | | | CCHCR1 | | | | | | A>G | | | |
| rs130076 | Chr6:31122482 | | | | CCHCR1 | | | | | | G>A | | | |
| rs130065 | Chr6:31122500 | | | | CCHCR1 | | | | | | GCC>ACC,GCT | | | |
| rs130075* | Chr6:31122502 | | | | CCHCR1 | | | | | | C>T | | | |
| rs144885162* | Chr6:31122564 | | | | CCHCR1 | | | | | | C>G | | | |

Note: dbSNP ID, data base SNP identification number at NCBI; Genome position, UCSC Genome Browser hg19 assembly; REF>ALT, reference base > alteration base; rs, reference SNP ID number; Chr, chromosome; ERAP1, endoplasmic reticulum aminopeptidase 1; NFKBIZ, NF-κB inhibitor zeta; TRAF3IP2, TRAF3 interacting protein 2; TNFAIP3, TNF alpha induced protein 3; TYK2, tyrosine kinase 2; IL17RA, IL-17 receptor A, CDSN, corneodesmosin; CCHCR1, coiled-coil alpha-helical rod protein 1.
